# The Impact of Histopathology on Medical Board Autopsies

**DOI:** 10.7759/cureus.78675

**Published:** 2025-02-07

**Authors:** Kumar Shubhendu, Sawan Mundri, Sanjay Kumar, Anand Kumar

**Affiliations:** 1 Department of Forensic Medicine and Toxicology, Rajendra Institute of Medical Sciences, Ranchi, IND

**Keywords:** custodial death, histopathology, medical board autopsies, nephritis, pneumonia

## Abstract

Background: Histopathological examination, although mostly limited to scenarios where the cause of death is not readily apparent during the autopsy, possesses substantial significance in medico-legal autopsies conducted by medical boards, as it provides essential insights into both the causation and circumstances contributing to death. Diverse viewpoints persist about how histopathology is utilized in the context of medico-legal autopsies. This study examines the significance of histopathology in elucidating particular causes of death in medico-legal autopsies conducted by a medical board of doctors, with a predominant proportion of cases of custodial deaths.

Materials and methods: The study concentrated on examining medico-legal autopsies, which were conducted by a panel of doctors from April 2015 to March 2020 in the Department of Forensic Medicine and Toxicology, Rajendra Institute of Medical Sciences, Ranchi, Jharkhand, that necessitated histopathological evaluation. Tissues were processed for histopathological examination in 177 (67.5%) cases during the five-year period among 262 medical board autopsies. The number of specimens obtained during each autopsy varied between one and six (mean = 03; mode = 02), resulting in a total of 511 specimens collected. The exhaustive analysis of various organs delineated in the initial autopsy documentation was contrasted with the histopathological findings articulated in the final report. Any discrepancies discovered were systematically classified, along with an organized categorization of differences between macroscopic and microscopic diagnoses and modifications to the ascertained cause of death following histopathological assessment. In situations where the cause of death remained unresolved during gross examination, the extent to which histopathological analysis contributed to the ultimate conclusions was evaluated concerning the detailed autopsy report.

Results: Pathological manifestations were predominantly detected within the gastrointestinal system. The most pronounced divergence between gross and histopathological assessments was identified in the specimens related to the cardiovascular system. The organ system that most frequently required microscopic examination to establish a conclusive diagnosis was the genitourinary system. The highest levels of concordance between macroscopic and microscopic evaluations were recorded within the nervous system. The definitive cause of death was either clarified or revised after microscopic evaluation in 33 (19.2%) cases, while the underlying indirect cause was clarified or altered in 7 (4.1%) cases. Histopathological findings corresponded with the established cause of death and provided additional insights in 65 (37.8%) cases, whereas they provided corroborative evidence in 66 (38.4%) cases. The pathological conditions frequently associated with altering the cause of death following histopathological evaluation encompassed pneumonia, tubular/interstitial nephritis, and pulmonary edema.

Conclusions: Histopathology constitutes a pivotal element of medico-legal autopsies, yielding indispensable information on the cause of death while also functioning as an educational instrument. Nevertheless, its effectiveness may fluctuate depending on the specific circumstances and death classification, emphasizing the necessity for a careful case-by-case methodology.

## Introduction

Histopathological examination of tissue samples is of utmost importance in medico-legal autopsies, functioning as a crucial means for revealing the cause of death, particularly in instances where external examination does not offer definitive conclusions. This analysis entails the microscopic evaluation of tissues to discern pathological processes, validate diagnoses, and reveal incidental observations that may remain undetected during autopsies and can, in various manners, influence the determination of the cause of death [1]. For example, histopathology is indispensable in recognizing conditions such as ischemic heart disease, which has experienced a notable increase in prevalence and frequently poses difficulties in cases of sudden mortality [2].

The relevance of histopathology encompasses a multitude of death classifications, inclusive of those resulting from unnatural causes, wherein it has the capacity to elucidate lesions that remain undetectable via macroscopic examination alone, as evidenced by investigations into adrenal gland pathologies [3]. Notwithstanding the ongoing discourse regarding its routine application, histopathology has been demonstrated to yield significant insights, particularly in cases of poisoning, where histopathological examination has proven crucial in identifying the pathological effects of toxins on various organs, thereby facilitating a more accurate determination of the cause of death [4].

In addition, examining histopathological specimens can expose illnesses that go unnoticed in a clinical context, which include tumors, infections, and inflammatory issues, thus enriching the knowledge of an individual's health profile and possible reasons for death [5,6], along with entailing the identification of incidental findings with profound implications for public health and preventive strategies [7]. For instance, identifying atherosclerosis in younger populations emphasizes the imperative for lifestyle interventions and preventive screening initiatives [5].

Moreover, histopathological analysis possesses the potential to either elucidate or substantiate the cause of death in a considerable proportion of cases, thereby emphasizing the significance of synergistic efforts between autopsy surgeons and clinical pathologists to facilitate precise diagnostic outcomes [8]. The application of specialized methodologies such as immunohistochemistry, albeit somewhat constrained, can further increase the diagnostic proficiency of histopathological assessments during autopsies [9]. Histopathological evaluation thus constitutes an essential element of medico-legal autopsies, furnishing vital information that bolsters the ascertainment of cause and manner of death, assists in the recognition of previously undiagnosed medical conditions, and enhances the overall comprehension of disease prevalence and mortality trends within the populace [10].

Not all medico-legal autopsies are consistently subjected to histopathological examination. Only those instances in which the cause of death is either not immediately obvious or apparent during gross autopsy are forwarded for histopathological evaluation [11]. In Europe, histological investigations are conducted in only about 50% of autopsies, with advanced techniques like molecular pathology being even less common [7]. Still, a range of views exists among scholars about how effective histopathological reviews are in legal settings, with some believing that conducting histopathological analyses in all legal situations, no matter the death's cause or manner, is redundant and ought to be limited to select cases rather than being a common approach [12]. In contrast, others support its prudent use [13,14].

While the significance of histopathology in the context of general autopsies is widely acknowledged, there exists a paucity of research specifically addressing its function and implications within medico-legal autopsies performed by medical boards, especially in instances of custodial deaths, which are characterized by intrinsic complexities and present distinct challenges such as the necessity for unequivocal and objective findings pertinent to legal proceedings, the susceptibility to external influences, and the pressing requirement for precise determination of the cause of death to ensure legal justice.

This study aims to evaluate the significance of histopathological examinations in refining or determining the cause of death in medico-legal autopsies performed by a medical board comprised of multiple experts (including autopsy surgeons alongside doctors from diverse specializations such as internal medicine, surgery, pathology, obstetrics, and gynecology, among others), with a particular emphasis on custodial deaths.

## Materials and methods

The present record-based observational study was executed within the Department of Forensic Medicine and Toxicology, Rajendra Institute of Medical Sciences, Ranchi, Jharkhand, encompassing all medico-legal autopsies performed by a medical board of doctors from April 2015 to March 2020. Records of the aforesaid autopsies that included post-mortem reports, histopathological examination reports, chemical analysis reports, and final cause of death reports were included in the study. Records deemed incomplete or ineligible, which posed challenges in interpretation, along with decomposed bodies, fetuses exhibiting congenital malformations, and autolyzed tissues identified during microscopic examination, were excluded from the analysis. All samples were diligently kept in a 10% neutral formaldehyde solution, and the histological sections complied with the processing standards of the Department of Pathology, Rajendra Institute of Medical Sciences, Ranchi, subsequently receiving staining with hematoxylin and eosin, along with various special stains according to particular needs. All examinations post-autopsy and their histopathological analysis were handled or guided by proficient pathologists. Ethical approval for the study was secured from the Institutional Ethics Committee of Rajendra Institute of Medical Sciences, as documented in memo no. 22, dated February 16, 2019.

A case report form was meticulously developed at the study's inception to systematically collect data after an extensive review of the existing literature. The data was systematically entered, and a template was generated, followed by the categorization of raw data to facilitate meaningful interpretation. Frequency tables and proportions were then generated to elucidate the descriptive distribution of the variables under investigation. False negative diagnoses that incorrectly indicated the absence of a disease and false positive diagnoses that incorrectly indicated the presence of a disease commonly responsible for altering the cause of death following histopathological examinations were examined. No identifiers were utilized during data acquisition, documentation, or analysis, ensuring the complete confidentiality of the collected information. No reviewer or external party has ever accessed the data. Throughout the data collection phase, the records were never left unattended and were securely stored in a locker after use.

The comprehensive characterization of distinct organs documented in the preliminary autopsy report was compared with the histopathological results presented in the final report, and any discrepancies identified were categorized employing criteria derived from Matkowski et al. [15] and Bernardi et al. [16] (Table 1).

**Table 1:** Classification of concordance between macroscopic and microscopic characterizations of organ pathology The criteria were modified from Matkowski et al. [15] and Bernardi et al. [16]. This source is under a Creative Commons license. BMJ Publishing Group Ltd. has the exclusive license for this source and permission regarding its use, specifically for the adaptation of the categorization methodology of the histopathology diagnosis as mentioned in the "Materials and Methods" section of the source, which has been duly taken from the BMJ Publishing Group Ltd.

Category	Class	Definition
Concordant	1a	Complete concurrence between macroscopic and microscopic analyses.
	1b	The microscopic analysis elucidated the preliminary diagnostic assessment.
Discordant	2	Discrepancy between macroscopic and microscopic evaluations
Histopathological analysis necessitated	3a	Indeterminate macroscopic assessment: histopathological analysis is essential for diagnostic purposes.
	3b	The gross examination yielded no significant findings; thus, histopathological analysis is essential for diagnostic purposes.

Variances between macroscopic and microscopic diagnoses were systematically classified. Amendments to the determined cause of death post-histopathological evaluation were likewise categorized according to the nature of the modification utilizing criteria adapted from Matkowski et al. [15] (Table 2).

**Table 2:** Classification of modifications to the cause of death subsequent to histopathological examination The criteria were modified from Matkowski et al. [15]. This source is under a Creative Commons license.

Class	Definition
I	Histopathological examination elucidated or modified the definitive cause of death.
II	Histopathology examination elucidated or modified the underlying indirect cause of death.
III	Histopathological findings align with the identified cause of death and offer supplementary insights.
IV	Histopathological findings are consistent with the etiological factors contributing to death and offer corroborative substantiation.
V	The histopathological analysis did not exhibit a definitive correlation with the established cause of death.

Specimens obtained from a variety of organs were meticulously examined. In instances where the cause of death remained indeterminate during gross inspection, the degree to which histopathology informed the final conclusions was assessed concerning the final autopsy report. A comparative analysis employing Cohen's kappa test was subsequently conducted between the recorded cause of death in provisional and final autopsy reports; the sensitivity and positive predictive value for each specific condition were then ascertained, in addition to conducting confidence interval assessments with five percent error for all sensitivities and positive predictive values using SPSS Statistics (IBM Corp. IBM SPSS Statistics for Windows. Armonk, NY: IBM Corp.).

## Results

Tissues were processed for histopathological examination in 177 (67.5%) cases during the five-year period among the 262 autopsies conducted by a medical board of doctors. Among the 177 cases examined, three involved bodies that had undergone significant decomposition or autolysis of tissues. In comparison, two additional cases were related to fetuses exhibiting congenital anomalies, necessitating their exclusion from further analysis.

The number of specimens obtained during each autopsy varied between one and six (mean = 03; mode = 02), culminating in 511 specimens. The majority of deaths were attributed to natural causes (86%) and custodial circumstances (86%). It is noteworthy that the coincidence of the figures for natural deaths and custodial deaths being identical does not imply that these categories were mutually exclusive (Table 3).

**Table 3:** General characteristics of autopsies performed under medical board in which tissues were sent for histopathological examination

Year-wise distribution of autopsies performed		
Month and year	Frequency (n=172)	Percentage (%)
April 2015 to December 2015	17	9.9
January 2016 to December 2016	34	19.8
January 2017 to December 2017	27	15.6
January 2018 to December 2018	44	25.6
January 2019 to December 2019	44	25.6
January 2020 to March 2020	6	3.5
Total	172	100
Distribution of autopsies performed according to the manner of death		
Manner of death		
Natural	148	86
Unnatural	24	14
Total	172	100
Distribution of autopsies performed according to the incarceration status		
Custodial deaths		
Yes	148	86
No	24	14
Total	172	100

Pathological manifestations were most commonly observed within the gastrointestinal system (25.1%), followed by respiratory (22.5%), genitourinary (22.3%), cardiovascular (13.5%), lymphatic (8.4%), and nervous (8.2%) systems (Table 4).

**Table 4:** Distribution of diagnoses according to major systems involved

Systems involved	Total diagnosis (n)	Percentage (%)
Nervous system	42	8.2
Cardiovascular system	69	13.5
Respiratory system	115	22.5
Gastrointestinal system	128	25.1
Genitourinary system	114	22.3
Lymphatic system	43	8.4
Total diagnoses	511	100

The most significant disparity between macroscopic and microscopic observations was observed in the specimens pertaining to the cardiovascular system (23.2%), followed by the lymphatic system (16.3%). The organ system that most often necessitated microscopic analysis to attain a definitive diagnosis was the genitourinary system (57%). The peak degrees of accord among macroscopic and microscopic observations were documented within the nervous system (42.9%), succeeded by the respiratory system (40.9%) and the gastrointestinal system (37.5%) (Table 5).

**Table 5:** Frequency of organ diagnoses (% of total) for each classification

Organ system	Total diagnosis, (n)	1a. Concordant, n (%)	1b. Concordant, n (%)	2. Discordant, n (%)	3a. Histology needed, n (%)	3b. Histology needed, n (%)
Nervous	42	7 (16.7)	11 (26.2)	6 (14.3)	4 (9.5)	14 (33.3)
Cardiovascular	69	0 (0.0)	18 (26.1)	16 (23.2)	14 (20.3)	21 (30.4)
Respiratory	115	3 (2.6)	44 (38.3)	18 (15.7)	23 (20)	27 (23.4)
Gastrointestinal	128	3 (2.3)	45 (35.2)	19 (14.8)	13 (10.2)	48 (37.5)
Genitourinary	114	2 (1.8)	38 (33.3)	9 (7.9)	18 (15.8)	47 (41.2)
Lymphatic	43	0 (0.0)	13 (30.2)	7 (16.3)	5 (11.6)	18 (41.9)

With regard to alterations in the cause of death following the histopathological examination, the final cause of death was either clarified or amended in 19.2% of instances. In comparison, the indirect cause of death was clarified or modified in 4.1% of instances. Histopathological observations matched the recognized cause of death and added extra perspectives in 37.8% of occurrences, aligning with the causal factors related to death, and yielded corroborative proof in 38.4% of occurrences. Only one case was noted where the histopathological evaluation did not reveal a definitive association with the recognized cause of death (Table 6).

**Table 6:** Documented alterations made to the cause of death following histopathology

Class	Frequency (n=172)	Percentage (%)
I	33	19.2
II	7	4.1
III	65	37.8
IV	66	38.4
V	1	0.5

The pathological conditions frequently implicated in modifying the cause of death after histopathological assessment included lobar/interstitial/bronchopneumonia, tubular/interstitial nephritis, and pulmonary edema, with a false negative diagnostic rate observed in 34 of the 43 specimens, while false positive diagnoses were evident in the remaining nine specimens upon gross examination of these conditions (Table 7).

**Table 7:** Diseases commonly responsible for altering the cause of death following histopathology Multiple findings in the same specimen. Excluding 14 cases of tuberculous pneumonia and 3 cases of renal tuberculosis confirmed by a Cartridge-Based Nucleic Acid Amplification Test (CBNAAT).

Disease	N (autopsy findings)	False negative	False positive
Pneumonia (lobar/interstitial/bronchopneumonia)	20	15	5
Nephritis (tubular/interstitial)	16	14	2
Pulmonary edema	7	5	2
Meningoencephalitis	4	3	1
Myocardial infarction	3	2	1
Pericarditis/myocarditis	3	3	0

The comparison of provisional autopsy reports with the final autopsy reports illuminated the metrics of true-positive, false-negative, and false-positive outcomes associated with these pathologies, thereby facilitating the calculation of sensitivity and positive predictive value. The calculated sensitivity for lobar, interstitial, and bronchopneumonia was 77.94%, with a confidence interval spanning from 66.24 to 87.10. It also possessed a positive predictive value calculated at 91.38%, with the confidence interval ranging from 90.33 to 92.32. In comparison, tubular and interstitial nephritis showed a sensitivity of 79.41%, with a confidence interval between 68.31 and 88.44, alongside a positive predictive value of 96.49%, with a confidence interval from 96.07 to 96.87. Meanwhile, pulmonary edema demonstrated a sensitivity of 66.67%, with a confidence interval ranging from 38.38 to 88.18, and a positive predictive value of 83.33%, with a confidence interval between 77.76 and 87.73 (Table 8).

**Table 8:** Sensitivity and PPV of the most common diseases found at autopsy across all classes of alteration Multiple findings in the same specimen. Excluding 14 cases of tuberculous pneumonia and 3 cases of renal tuberculosis confirmed by a Cartridge-Based Nucleic Acid Amplification Test (CBNAAT). CI: confidence interval, PPV: positive predictive value

Disease	N (autopsy findings)	True positive	False negative	False positive	Sensitivity (%)	95% CI	PPV (%)	95% CI
Pneumonia (lobar/interstitial/bronchopneumonia)	73	53	15	5	77.94	66.24-87.10	91.38	90.33-92.32
Nephritis (tubular/interstitial)	71	55	14	2	79.71	68.31-88.44	96.49	96.07-96.87
Pulmonary edema	17	10	5	2	66.67	38.38-88.18	83.33	77.76-87.73

The viscera, specifically the stomach along with its contents, a segment of the small intestine, portions of the liver and spleen, as well as half of each kidney, were preserved in a supersaturated solution of common salt for the purpose of toxicological examination in a total of 16 cases, out of which methyl alcohol was identified in 6 instances associated with hooch-related fatalities, with the microscopic observations aligning with the characteristics of methyl alcohol poisoning. In the remaining 10 cases, the results of the chemical analysis were negative for the presence of any metallic, alkaloidal, pesticidal, volatile, or non-volatile toxins. In these instances, the microscopic findings provided insight into or refined the understanding of the underlying causes of death.

## Discussion

The impact of histopathology in medico-legal autopsies remains a focal point of substantial dialogue, with numerous viewpoints about its critical nature and success in explaining the cause of death. Histopathology is frequently utilized to augment the conclusions drawn from a conventional autopsy, especially in scenarios where the cause of death is not readily apparent from gross anatomical inspection. Many studies emphasize the prospective advantages of histopathology within the realm of medico-legal autopsies [13,14]. For instance, the analysis of tissue characteristics can corroborate or refute observable findings, thereby contributing to a more nuanced comprehension of the factors leading to death.

However, the significance of regular histopathological assessments in all medico-legal autopsies is a topic of contention. Certain research studies indicate that histopathology does not significantly influence the ascertainment of the cause of death in specific categories of cases, particularly when the uncertainty regarding the cause of death is minimal during medico-legal autopsy [4]. This suggests that, although histopathology may enhance diagnostic certainty, its significance could be restricted in instances where the cause of death is already evident from macroscopic observations. Furthermore, integrating histopathology into medico-legal autopsies encounters various challenges, including time constraints, issues related to tissue preservation, a deficiency in standardization, exorbitant costs, communication barriers among specialists, and pertinent legal and ethical dilemmas. Additionally, the procedure may be resource-intensive, potentially putting additional strain on autopsy surgeons, clinical pathologists, and law enforcement organizations.

Notwithstanding these obstacles, there exists a prevailing agreement among numerous specialists that histopathology is an essential instrument in medico-legal autopsies. It yields objective and reproducible evidence that can enhance the conclusions drawn from a macroscopic autopsy, a factor of particular significance in judicial contexts where the admissibility of such evidence is paramount [17-18]. The medical boards in the current research to investigate this specific utility of histopathology comprise practitioners from diverse specialties, presided over by an autopsy surgeon. The spectrum of cases typically designated for autopsy by medical boards included custodial deaths, alleged medical negligence deaths, hooch tragedies, casualties from landmine explosions, and mass homicide-suicide occurrences, alongside cases that exacerbated societal tensions and posed a risk of fracturing the social fabric, thereby necessitating the reinforcement of the assertion that “justice has been done,” concerning the autopsy. The same is analogous to an autopsy conducted by a medical board of doctors from various specialties converging on the determination of the cause of death in such cases.

The number of specimens acquired during each autopsy in the current study ranged from one to six. The majority of deaths were ascribed to natural causes occurring within custodial environments. Pathological manifestations were predominantly detected within the gastrointestinal system, followed by the respiratory and genitourinary systems, contrasting with findings from certain prior studies [15,19-20]. The prevalence of those in incarceration may explain this phenomenon, suggesting a multifaceted interaction between human physiology, the molecular processes of aging, sociocultural contexts, and the implications of environmental health.

The divergence between macroscopic and microscopic findings represents a critical concern that influences the precision and dependability of medico-legal autopsies. This variance emerges from the distinctions in visibility and interpretability of findings across varying scales, which affect the elucidation of the cause and manner of death. The most pronounced discrepancy between macroscopic and microscopic findings was identified in specimens associated with the cardiovascular system, followed by the lymphatic system, which contrasts with the findings of other researchers [14-16,19], whose studies indicated that discrepancies were predominantly observed in lung specimens, succeeded by those of the liver. The organ system that frequently required microscopic examination to achieve a conclusive diagnosis was the genitourinary system, aligning with the investigations conducted by Matkowski et al. [15] and Bernardi et al. [16]. The peak agreement noted between macroscopic and microscopic findings occurred in the nervous, respiratory, and gastrointestinal systems. In the investigations of Matkowski et al. [15] and Bernardi et al. [16], convergence was similarly most prevalent in specimens derived from the brain.

Histopathology serves as an important factor in assessing the cause of death, especially when physical pathological analyses fall short of clarity. In instances of abrupt or unforeseen deaths, histopathology plays a pivotal role in elucidating the precise cause of death, which is imperative for both legal and medical considerations [21]. Concerning alterations in the causes for death following histopathological investigations, the final cause was either made clear or adjusted in 19.2% of occurrences. In contrast, the indirect cause of death was elucidated or modified in 4.1% of occurrences. Histopathological findings corresponded with the established cause of death. They provided additional insights in 37.8% of instances and aligned with the causal determinants associated with death, yielding corroborative evidence in 38.4% of cases. These statistical figures are inconsistent with the observations reported by Matkowski et al. [15], whose research indicated proportions of 45.4%, 7.1%, 9.2%, and 27.7%, respectively.

The pathological entities that are often associated with the modification of death attribution after histopathological examination encompassed lobar/interstitial/bronchopneumonia, tubular/interstitial nephritis, and pulmonary edema, with a false negative diagnostic rate identified in 34 out of the 43 specimens, whereas false positive findings were apparent in the remaining nine specimens upon microscopic examination. Nonetheless, Matkowski et al. [15] reported that pneumonia, in conjunction with primary malignancy, represented the most prevalent diseases accountable for the alteration of the cause of death following histopathological analysis.

The comparative analysis of autopsy findings alongside the definitive documentation of the cause of death enabled the computation of sensitivity and positive predictive value. Determining the cause of death due to pulmonary edema via macroscopic examination exhibited the lowest sensitivity at 66.67% (95% CI = 38.38-88.18). Precisely delineating pulmonary pathology from macroscopic examination in isolation is frequently challenging, resulting in a limited clinicopathological correlation [22-24].

Histopathological examination augments various forensic methodologies, including toxicological analyses, facilitating a holistic evaluation [18]. This assertion is further substantiated in the current investigation, wherein the viscera were meticulously preserved for toxicological analysis across 16 cases, during which the microscopic observations yielded significant insights or enhanced comprehension regarding the fundamental etiological factors contributing to death.

Concerning the phenomenon of natural deaths, the research conducted by various scholars [11,13-14,24] corroborates the conclusions of the present study, asserting that histopathological analysis of tissues in the context of a medico-legal autopsy plays a pivotal role in elucidating the final cause of death. Conversely, this conclusion contradicts the findings put forth by some other scholars [12,20,25].

The paper highlights the significance of synergistic collaborations between autopsy surgeons and clinical pathologists to optimize the diagnostic advantages of histopathology, as evidenced by its capacity to alter or validate the cause of death in a considerable proportion of medico-legal cases. Consequently, histopathology is an indispensable instrument in forensic medicine, augmenting the precision and dependability of cause-of-death evaluations through meticulous microscopic examination. This can yield significant ramifications in legal and forensic contexts, particularly in alleged malpractice, negligence, or criminal inquiries. Precise post-mortem evaluations can substantially affect judicial conclusions, especially in scenarios where the causation of death is disputed (e.g., fatalities resulting from medical mismanagement, homicide, or ambiguous complications arising from medical interventions) and prior clinical evaluations were erroneous or insufficient. In the context of insurance claims, evaluations of disability, or litigation about wrongful death, histopathological evidence could furnish supplementary documentation and facilitate more precise death certificates, thereby influencing financial, legal, and compensatory determinations.

This research also suggests that histopathological evaluation is integral in augmenting diagnostic precision. Within clinical contexts, this underlines the significance of medico-legal autopsies, not solely for elucidating the cause of death but also for advancing diagnostic precision in living individuals. The study accentuates how histopathological revelations frequently elucidated conditions that were either subtle or not readily apparent during preliminary clinical assessments. Identifying nuanced or previously undiagnosed conditions may enhance epidemiological monitoring and facilitate early, targeted interventions within high-risk demographic groups.

Although microscopic evaluation can yield essential insights, its systematic application in all medico-legal autopsies may not be warranted when considering cost-benefit assessments and the associated workload on clinical pathologists. The decision to employ microscopic analysis should be guided by the specific contextual factors unique to each situation, balancing the imperative for a comprehensive examination with practical considerations such as financial implications and time limitations.

While histopathological evaluations yield essential insights regarding the cause of death, they are not without limitations. The intricate nature of pathological states and the possibility of diagnostic inaccuracies emphasize the necessity for thorough autopsy protocols and the amalgamation of clinical and pathological information. Furthermore, the variability observed in histopathological results across diverse conditions accentuates the significance of context-specific assessments. Though faced with these difficulties, histopathology is an invaluable asset in forensic and clinical pathology, granting a deep understanding of disease mechanisms and mortality factors.

Limitations

The cases analyzed were conducted more than four years ago, with certain instances undergoing autopsy during the COVID-19 pandemic's initial phase (from December 2019 to March 2020). The ramifications of SARS-CoV-2 on diverse organ systems throughout this timeframe have not been considered in this research due to the unavailability of data related to the COVID-19 status of the deceased. Nevertheless, the inferences drawn remain relevant, as there has been no significant alteration in histopathological methodologies during the elapsed period.

Data was amassed from autopsies conducted by a panel of medical professionals, wherein tissue specimens were dispatched for microscopic analysis, based on the judgment, albeit in agreement with the board members, thereby introducing a discernible selection bias, primarily minimized by employing the prevalent standard autopsy protocols observed in nearly all medical institutions across India.

The research does not acknowledge the potential role of aspects like concurrent illnesses, choices in lifestyle, or pharmaceutical interventions on organ ailments, which could have produced a more integrated view of the findings. In addition, the investigation solely centered on autopsy specimens sourced from a particular tertiary care institution, which might not depict the attributes of the broader population, limiting generalizability; moreover, it does not consider the potential issues or limitations related to the interpretation of microscopic findings, including observer differences or the influence of postmortem modifications on tissue anatomy, along with issues related to the exclusion of autolyzed tissues and decomposed bodies that can lead to underreporting of specific associated pathological findings.

## Conclusions

While the customary application of histopathology in all medico-legal autopsies may not invariably be deemed essential, its function in furnishing comprehensive, corroborative evidence renders it an invaluable element of forensic medicine. The determination to utilize histopathology ought to be informed by the particulars of each individual case, weighing the imperative for an exhaustive investigation against the pragmatic considerations of resource management. The formulation of guidelines, predicated on the insights of all relevant stakeholders, to delineate cases wherein histopathology can yield substantial contributions could enhance its application within forensic practice. The interrelation between macroscopic and microscopic examinations necessitates an integrative approach to autopsy interpretation, wherein both tiers of analysis are synthesized to foster a unified comprehension of the causative factors and manner of death. This multifaceted methodology is crucial for addressing the intricacies of each case and guaranteeing that the conclusions derived are substantiated and representative of the fundamental pathophysiological mechanisms. Ultimately, histopathological assessment persists as a pivotal complement to gross autopsy findings, augmenting the precision and dependability of determinations regarding the cause of death in the realm of forensic medicine.

Further prospective studies can be undertaken by including those cases where histopathology was not requested in medical board autopsies, preferably employing a multi-centric inter-departmental approach to ensure a larger sample size using insights from autopsy surgeons and pathologists from various medical institutions. The likely observation bias by pathologists and the feasibility of direct supervision of autopsy surgeons in histopathology analysis should also be factored in in further studies, along with investigating the cost-effectiveness of histopathology in different settings. Further research can also be conducted to compare routine autopsies and board cases to analyze relevant factors in corroboration with the clinical data by collaborating with other departments of medical institutions (in which the patients were admitted before their demise). In medico-legal autopsies, advanced techniques such as immunohistochemistry and molecular pathology should also be explored.
